# Cognitive Bias in the Management of a Critically Ill 29-Year-Old Patient

**DOI:** 10.7759/cureus.39314

**Published:** 2023-05-21

**Authors:** Jessica Vittorelli, Jenna Cacchillo, Michael McCool, Andrew McCague

**Affiliations:** 1 Emergency Medicine, Desert Regional Medical Center, Palm Springs, USA; 2 Trauma and Acute Care Surgery, Desert Regional Medical Center, Palm Springs, USA

**Keywords:** diagnostic momentum, anchoring bias, septic shock, abdominal compartment syndrome, cognitive bias

## Abstract

Cognitive bias is a significant issue in the management of critically ill patients. Often patients cannot communicate due to illness or mechanical ventilation, making history-taking difficult. Here we present a case where cognitive bias led the clinical team to treat the wrong diagnosis until the patient was in extremis.

We present a 29-year-old otherwise healthy female who initially presented to an outside facility with severe abdominal pain and hypotension. Due to a history of medical abortion two weeks prior, the patient was initially diagnosed with sepsis due to retained products of conception. Following a dilation and curettage that revealed no retained POC and worsening of the patient's symptoms, the patient was transferred to our facility for higher care. Over five additional days, the patient had a significantly worsening clinical picture before new diagnoses such as abdominal compartment syndrome, necrotic bowel, and adverse effects from diet pill cleanse were considered and acted upon. The patient ultimately suffered abdominal and bilateral lower extremity compartment syndrome leading to colectomy and bilateral below-the-knee amputations.

As clinicians, we must provide the best care possible and reduce patient suffering. Cognitive bias is something that all clinicians must be aware of and learn to manage. Failure to be aware of one's cognitive bias puts the patient at risk and can be harmful. This case illustrates just how detrimental cognitive bias and misdiagnoses can be.

## Introduction

Cognitive bias has been identified as a significant problem in U.S. hospitals. A 2016 Joint Commission publication reported that diagnostic errors are associated with 6-17 percent of hospital adverse events, and 28 percent of diagnostic errors have been attributed to cognitive error [1]. Cognitive error is common in clinical practice, and up to 75% of errors in internal medicine practice are thought to be cognitive in origin [2]. Reflecting on personal errors, doctors identify cognitive factors in 30% of errors in the emergency department and 42% in internal medicine wards [2]. Furthermore, these errors cause high costs as malpractice payouts. Here we present a case of a 29-year-old female who suffered misdiagnoses attributed to cognitive bias.

## Case presentation

This is a case of a 29-year-old female (G4P2) with a recent history of COVID-19 and medical abortion who presented to an outside hospital complaining of abdominal pain. She reported a past medical history of pancreatic cancer at 9 years old and asthma. She had a surgical history significant for the Whipple procedure and Cesarean Section. She denied allergies or home medications. 

She complained of a two-week history of lower abdominal pain and vaginal bleeding at her initial presentation. She reported recently using an over-the-counter colon cleanse' after having her abortion. She said she took double the dose, hoping for a larger effect. She worked at a small hospital about 60 miles from our facility. She presented there two days before transfer to our facility. She initially complained of abdominal pain and vaginal bleeding. She was admitted and later transferred to their ICU when she became hypotensive. A CT scan and Ultrasound showed an endometrial stripe concerning endometritis. She was started on antibiotics and underwent a bedside dilation and curettage, which was negative. As she continued to decline, she was transferred to our facility for higher care. 

 Upon arrival at our facility, her exam was significant for tachycardia, decreased breath sounds, diffuse abdominal pain, and bilateral edema of her lower extremities. She presented with a WBC of 58.3 103/mm3, Hemoglobin 14 g/dL, Hematocrit 44.7, Platelets 428 103/µL, Lactic Acid 3.0 mmol/L, Sodium 127 mmol/L, Potassium 3.9 mmol/L, Chloride 105 mmol/L, Bicarbonate 15 mmol/L, Glucose 353 md/dL, Blood Urine Nitrogen 33 mg/dL, and Creatinine 1.5mg/dL. 

**Figure 1:**
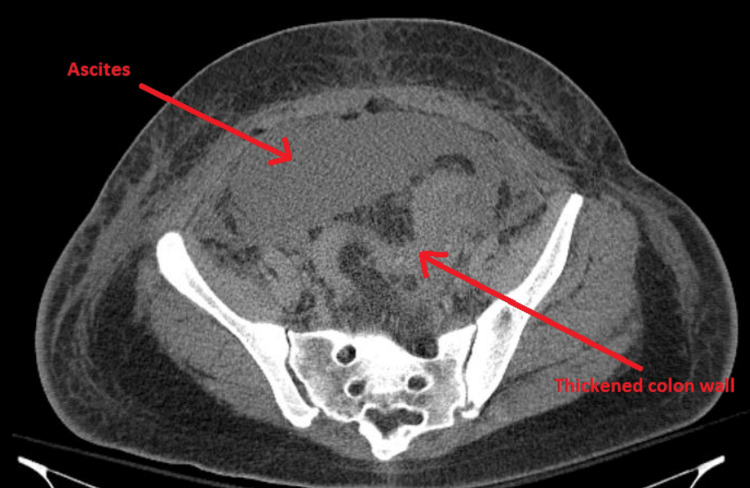
CT of abdomen/pelvis. Arrows represent thickened colon wall and ascites.

 A CT Abdomen/Pelvis reported large bilateral pleural effusions, moderate ascites, diffuse anasarca, and thickened colon wall, possibly due to colitis (Figure 1). 

The patient was admitted to the ICU and started on broad-spectrum antibiotics, including Clindamycin, Gentamicin, and Ampicillin, for concerns of endometritis. She continued to decompensate, requiring vasopressors. She went into renal failure and was started on hemodialysis. She was then intubated on hospital day number four. On hospital day number five, she was found to be in multisystem organ failure. The family was approached and offered a withdrawal of care. Acute Care Surgery was consulted just before the family decided on terminal extubation. She was found to have abdominal compartment syndrome. She was taken to the operating room emergently for an exploratory laparotomy and sigmoidectomy and left in discontinuity for an ischemic colon. Bilateral chest tubes were placed for significant pleural effusions concerning tension physiology. Her abdomen was left open for continued resuscitation. Postoperatively she improved significantly. She was on four vasopressors pre-operatively and was off all pressors when she returned to the ICU. On exam the following day, she was found to have pulseless lower extremities. She was returned to the OR for bilateral lower extremity fasciotomies for extremity compartment syndrome. She remained intubated and resuscitated. She later returned to the operating room for an additional exploratory laparotomy, where a colostomy was created and her abdomen closed. She was then transferred out of the ICU. Her feet did not recover, and she had significant ischemia requiring bilateral lower extremity amputations. She was eventually discharged to rehab on hospital day number forty-five. 

## Discussion

The above case represents a growing concern for cognitive bias in healthcare decision-making. Cognitive bias has been recognized within the medical community since the 1970s, and research has been mainly limited to the military, economics, and business [2]. In internal medicine, up to 75% of errors are cognitive in origin [3]. It is likely that most, if not all, clinical decision-makers are at risk of error due to bias [2].

In this particular patient, the initial diagnosis of endometritis was accepted on her arrival (anchoring bias) and not challenged. She developed septic shock for which she was treated. Other diagnoses were not initially entertained, including colon ischemia, abdominal compartment syndrome, tension hydro-thoraces, and lower extremity compartment syndrome. Her care suffered from diagnostic momentum bias as the early diagnoses were only minimally worked up. Later in her care, a premature closure bias seemed to doom her course. Once a new perspective was introduced to her care via an Acute Care Surgery, consult additional diagnostic potentials were entertained. 

Cognitive biases are increasingly recognized as contributors to patient safety events. Cognitive biases are flaws or distortions in judgment and decision-making [1]. Table 1 below lists only a limited subset of cognitive bias types seen in clinical practice. 

**Table 1:** Types of Cognitive Bias in Clinical Practice. Adapted from reference [1].

Bias Type	Description
Anchoring bias	Relying on initial information/impressions and not deviating (anchoring) from this despite new information becoming available.
Ascertainment bias	Making decisions based on prior expectations (stereotypes, gender, etc.). “Frequent flyers” or patients who repeatedly use the call bell may lead staff to expect this behavior.
Availability bias	Favoring a diagnosis based on the ease at which similar examples can be obtained (familiar diagnosis, memorable patients, common/recent events).
Conformation bias	Searching for information that confirms an initial impression rather than looking for evidence to disprove an impression.
Diagnostic momentum	Once a patient is given a diagnosis, momentum reduces the likelihood of additional alternative diagnoses being worked up or entertained.
Framing effect	The way in which information is presented or framed can impact future decisions.
Search satisficing/premature closure	Stop looking for other findings after something has been identified. Accepting a diagnosis before all information has resulted.

As encouraged by the 2016 Joint Commission publication, active efforts need to be pursued within hospitals and other healthcare settings to help medical teams navigate the 'human factors' that affect patient safety [2]. 

Many approaches have been ensured to assess and evaluate clinical cognitive errors [3]. Preparing education programs to enhance the knowledge and awareness of cognitive biases can be started [1]. Discussing these 'human factors' in Morbidity and Mortality and Peer Review meetings can bring a new light to a case review [1]. Enhancing provisional reasoning, critical thinking, and decision-making skills is challenging, but several examples have shown effectiveness [1]. A 'diagnostic timeout' can facilitate an open and active discussion around alternative diagnoses [1]. Simulation exercises focusing on providing feedback around diagnostic decision-making can improve one's insight into their own bias. A review of the work-system conditions is imperative. Fatigue and long working hours can impact cognitive bias [1]. Multiple interruptions, such as frequent phone calls while working up a patient, can affect decision-making [1].

Clinician awareness of cognitive bias can help prevent the delays in diagnosis seen within the case presented here. 

## Conclusions

As clinicians, we must provide the best care possible and reduce patient suffering. Cognition bias is something that all clinicians must be aware of and learn to manage. Failure to be aware of one's cognitive bias puts patients at risk and can harm patients. This case illustrates just how detrimental cognitive bias and misdiagnoses can be.
